# Genome-wide sequencing identified extrachromosomal circular DNA as a transcription factor-binding motif of the senescence genes that govern replicative senescence in human mesenchymal stem cells

**DOI:** 10.3389/fncel.2024.1421342

**Published:** 2024-08-02

**Authors:** Wei Yang, Wei Ji, Boyu Liao, Zhongbo Li, Jian Wang, Haishu Lin, Jingbo Wang, Qian He

**Affiliations:** ^1^School of Food and Drug, Shenzhen Polytechnic University, Shenzhen, China; ^2^School of Life Science and Technology, Changchun University of Science and Technology, Changchun, China; ^3^College of Pharmacy, Shenzhen Technology University, Shenzhen, China; ^4^School of Pharmaceutical Sciences, Health Science Center, Shenzhen University, Shenzhen, China

**Keywords:** biomarker, extrachromosomal circular DNAs, mesenchymal stem cells, senescence, transcription factors

## Abstract

**Introduction:**

Mesenchymal stem cells (MSCs) have long been postulated as an important source cell in regenerative medicine. During subculture expansion, mesenchymal stem cell (MSC) senescence diminishes their multi-differentiation capabilities, leading to a loss of therapeutic potential. Up to date, the extrachromosomal circular DNAs (eccDNAs) have been demonstrated to be involved in senescence but the roles of eccDNAs during MSC.

**Methods:**

Here we explored eccDNA profiles in human bone marrow MSCs (BM-MSCs). EccDNA and mRNA was purified and sequenced, followed by quantification and functional annotation. Moreover, we mapped our datasets with the downloading enhancer and transcription factor-regulated genes to explore the potential role of eccDNAs.

**Results:**

Sequentially, gene annotation analysis revealed that the majority of eccDNA were mapped in the intron regions with limited BM-MSC enhancer overlaps. We discovered that these eccDNA motifs in senescent BMSCs acted as motifs for binding transcription factors (TFs) of senescence-related genes.

**Discussion:**

These findings are highly significant for identifying biomarkers of senescence and therapeutic targets in mesenchymal stem cells (MSCs) for future clinical applications. The potential of eccDNA as a stable therapeutic target for senescence-related disorders warrants further investigation, particularly exploring chemically synthesized eccDNAs as transcription factor regulatory elements to reverse cellular senescence.

## Introduction

Numerous diseases and toxic factors can impair cellular and organic functioning in animals, emphasizing the crucial role of adult stem cells in homeostasis and tissue repair (Hwang et al., 2009). While organ transplantation was historically the only option for tissue healing, stem cell transplantation is currently a viable route in regenerative medicine (Hu and Li, 2018). MSCs have gained popularity due to their self-renewal, differentiation abilities, and paracrine capabilities, making them important candidates for tissue regeneration (Keshtkar et al., 2018; Lin et al., 2019). Their successful application has spread to a wide range of conditions, including liver damage, diabetes, and degenerative diseases (Chen et al., 2022).

Despite the potential of MSC-based therapy, senescence and senescence-related processes can restrict its efficacy (Wei et al., 2013). MSCs’ regenerative capacity is commonly acknowledged to decline with systemic age, perhaps contributing to senescence-related diseases (Ameh et al., 2022). Senescence MSCs, for example, prefer adipogenesis over osteogenesis, which reduces their bone-building capability (Marędziak et al., 2016). Furthermore, adult adipose-derived MSCs from older donors produce higher levels of pro-inflammatory cytokines, which may affect their immunomodulatory function (O’Hagan-Wong et al., 2016; Kizilay Mancini et al., 2017). Furthermore, MSCs’ replicative senescence limits their proliferative potential, which is critical for therapeutic applications that require large numbers of cells (Severino et al., 2013; Zhu et al., 2021). Understanding MSC senescence pathways is critical for improving therapeutic efficacy.

Senescence is characterized by a decrease in normal cell proliferative potential (Weng et al., 2022). Prolonged MSC culture induces DNA damage responses and repair mechanisms, eventually leading to cellular senescence (banimohamad-shotorbani et al., 2020). Notably, researchers have discovered the production of semi-stable eccDNA via DNA damage repair pathways, which could have ramifications for cellular function and aging (Wang et al., 2021). eccDNA is a form of double-stranded, non-structured circular DNA that exists outside the cell nucleus. It encompasses a variety of repetitive sequences, including long interspersed nuclear elements (LINEs), short interspersed nuclear elements (SINEs), satellite DNA, long terminal repeats (LTRs), and gene segments (Qiu et al., 2021). EccDNA inherits in a non-chromosomal manner and is unequally partitioned into daughter cells due to the lack of centromeres (Turner et al., 2017). As a result, eccDNA levels vary rapidly with the environment and are involved in a range of biological processes, including cancer, drug resistance, and senescence (Cohen et al., 2006; Cao et al., 2021). Chronic eccDNA has been associated with chromatin imbalance, genomic instability, and other abnormalities that could lead to illness or death (Zuo et al., 2021). Previous studies and reviews have stated that the related mechanisms of eccDNA include gene amplification, aggregation to operate as a hub to promote transcription, and immune system activation via linked pathways (Paulsen et al., 2021; Wang et al., 2021; Li et al., 2022). However, the role of eccDNA in the regulation of human MSCs is unknown.

Using sequencing data from BM-MSCs, we investigate the link between eccDNA and senescence-related genes in this study. Our findings are intriguing because they imply that eccDNA acts as a motif, binding TFs to control gene expression. These findings have the potential to help researchers better understand the self-renewal capacities and aging trajectories of human MSCs, perhaps opening up therapeutic approaches to combat cellular senescence.

## Materials and methods

### Cell culture, eccDNA purification, and sequencing

Two distinct human BM-MSCs named (HUXMA-01001) from the second passage were obtained from Cyagen and maintained in the MesenCult™-ACF Plus Culture Kit (STEMCELL Technologies, # 05448). BM-MSCs were passaged every 3–7 days to acquire Y51 and Y52 samples (group Y5, representing young cells) of the fifth passage, and an additional 10 passages were performed to generate Y151 and Y152 samples (group Y15, representing senescence cells). Each group was divided into two halves, one for Circle-Seq and the other for mRNA-Seq. The Circle-Seq eccDNA method for budding yeast (Møller et al., 2016) was used to purify the eccDNA fragments from cells, primarily using the Plasmid Mini AX kit for eccDNA enrichment. In summary, cells were lysed with Proteinase K (>0.1 U/μL, Life Technologies), and genomic DNA was extracted with Qiagen kits. The whole DNA was alkaline treated to separate chromosomal DNA, lipids, and protein by rapid DNA denaturing-renaturing, then column chromatography on an ion exchange membrane column (Plasmid Mini AX; A&A Biotechnology). Exonuclease (Plasmid-Safe ATP-dependent DNase, Epicentre) was used to remove the remaining linear DNA, which was aided by the rare-cutting endonuclease MssI, which digested mitochondrial circular DNA (mtDNA, 16 kb) and created more accessible DNA ends for exonuclease. EccDNA-enriched samples were used as a template for phi29 polymerase (REPLI-g Midi Kit) amplifying DNA reactions. Amplified circular DNA was cleaned with AMPure XP beads and sonicated (Bioruptor) to achieve an average fragment size of 200–300 bp. Libraries for next-generation sequencing were produced using the NEBNext Ultra DNA Library Kit for Illumina according to the manufacturer’s protocol (New England Biolabs) and sequenced on Illumina NovaSeq 6000 using PE150.

### mRNA purification and sequencing

Total RNA of cells was extracted using the TRIzol^TM^ reagent (Invitrogen, Carlsbad, CA, USA) as directed by the manufacturer’s instructions. The RNA concentrations were measured with the Qubit® RNA Assay Kit in the Qubit® 2.0 Fluorometer (Life Technologies, CA, USA). The NanoPhotometer® spectrophotometer (IMPLEN, CA, USA) and the RNA Nano 6000 Assay Kit of the Bioanalyzer 2100 system (Agilent Technologies, CA, USA) were used to assess RNA purity and integrity, respectively. rRNA was removed by Epicentre Ribo-zero™ rRNA Removal Kit (Epicentre, USA). Epicentre Ribo-zero^TM^ rRNA Removal Kit (Epicentre, USA) was used to remove the rRNA. Ethanol precipitation was used to clean up the rRNA-free residue. The NEBNext® Ultra^TM^ Directional RNA Library Prep Kit for Illumina (NEB, USA) was then used to create RNA-seq libraries. The libraries were sequenced using 150 bp paired-end mode on the Illumina NovaSeq 6000 platform.

### Quality control of eccDNA raw data and mRNA raw data and reads mapping statistics

Trimmomatic (Bolger et al., 2014) was used to filter out low-quality reads and sequences containing ploy-N and adaptor sequences from raw sequencing data from the Illumina NovaSeq 6000. BWA-MEM (Jung and Han, 2022) was used to align clean reads from the eccDNA database to the human reference genome (version hg38/GRCh38). Clean mRNA-Seq reads were aligned to the same genome as above using the TopHat v2.1.1 (Langmead et al., 2009) aligner. Samtools (Danecek et al., 2021) was used to sort, index, and statistically analyze these Bam files.

### The analysis of eccDNA and mRNA quantification and functional annotation

The BWA-MEM-matched reads were fed into the Circle-Map workflow (Prada-Luengo et al., 2019) to detect the circular DNA, and the split was utilized to screen for eccDNA. Each eccDNA contained at least one split read, which was chosen for further investigation. BEDTools v2.27.1 (Quinlan and Hall, 2010) was used to annotate eccDNA genes. For motif analysis, the MEME suite (Bailey et al., 2015) was used with the default parameters.

The TopHat-matched reads were processed with StringTie v2.2.1 (Shumate et al., 2022) to calculate the read counts of genes summarized from the mRNA level. The gene type was annotated with GENOME database (v30) (https://www.gencodegenes.org/human/release_30.html).

### Gene differential expression

DESeq2 (Love et al., 2014), with default parameters, was used for gene differential expression analysis. Genes with an absolute value of log2FoldChange >1 and a *p*-value ≤0.01 were considered substantially enriched. The pheatmap library of Rscrip^2^ was used to generate heatmaps of substantially enriched genes.

### The analysis of functional annotation and PPI

Based on the Metascape website (Szklarczyk et al., 2019), the Kyoto Encyclopedia of Genes and Genomes (KEGG) pathway analysis and Gene Ontology (GO) analysis were carried out, which classified genes into hierarchical categories and exposed the gene regulatory network using a database of the most recent biological processes. To control the false discovery rate (FDR < =0.05) and identify significant KEGG pathways and GO keywords, the p-value was corrected using the Benjamini and Hochberg approach.

PPI analysis with relative GO/KEGG annotation of subset gene sets was performed using the STRING website (Shannon et al., 2003). The following networks were created using Cytoscape v3.9.0 (Chin et al., 2014), which was used to discover hub modules (cytoHubba) and hub subnetworks (MCODE) (Han et al., 2018).

### The downloading of the datasets about enhancer and regulated genes by TF

The four BED files from ChIP-Seq on H3K27ac based on BM-MSCs (GSM1112792, GSM1112793, GSM1112797, and GSM1112798) were downloaded from NCBI. The intervals shared by the four datasets were used to create the final enhancer database through a customized pipeline. The regulated gene sets by known human TF were downloaded from Transcriptional Regulatory Relationships Unraveled by Sentence-based Text Mining (TRRUST) version2 website ⑯.

## Results

### Genome-wide sequencing of eccDNAs in BMSCs

**Figure 1:**
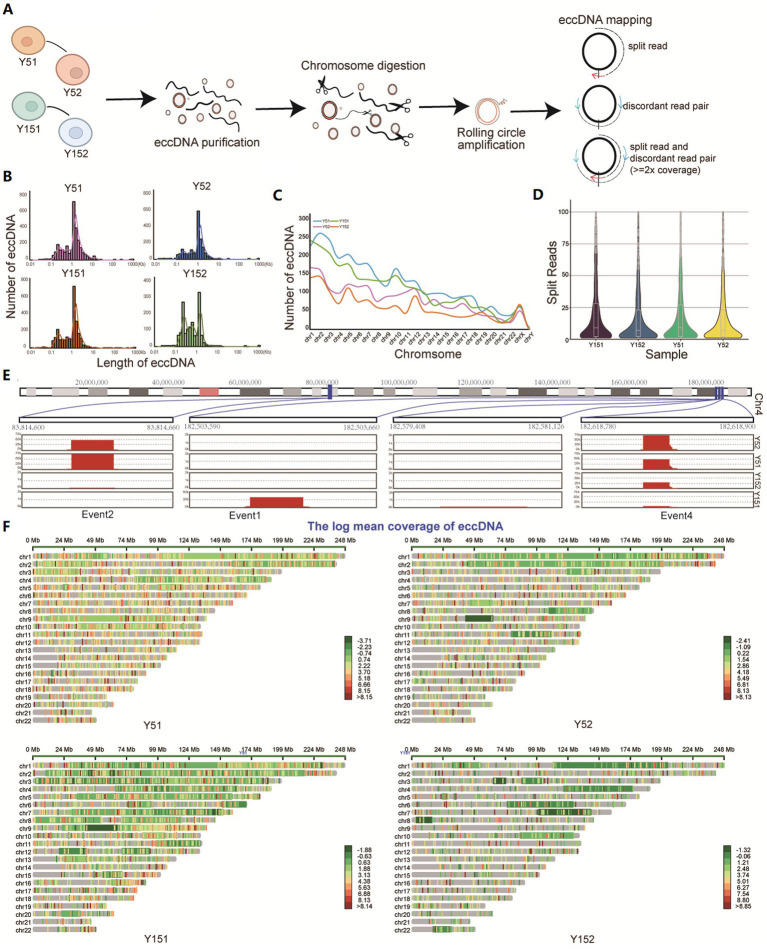
Identification of eccDNA in cells at different cultural stages. **(A)** Cells from different cultural stages were collected and lysed from Y5 and Y15; DNA fragments were purified, amplified, sequenced, and analyzed. All mappable reads were analyzed. **(B)** Length distribution of eccDNA identified in all samples. **(C)** Number distribution of eccDNA on 23 chromosomes in four samples. **(D)** The reads count of split reads supporting eccDNA fragments. **(E)** Distribution of some eccDNAs on chromosome 4. **(F)** Overall chromosomal distribution of eccDNAs across the genome.

In the study, eccDNA sequencing was performed on representative young cells (group Y5, comprised of Y51 and Y52 samples) and senescence cells (group Y15, encompassing Y151 and Y152 samples). The mapped reads were evaluated after stringent read filtering (Figure 1A). Detection of eccDNAs relied on abnormally mapped reads, which included split reads (reads mapped to two distinct locations on the reference genome) and discordant reads (paired reads facing outward on the reference genome) (Kang et al., 2023; Figure 1A). eccDNA from all four samples had bimodal size distributions with summits at approximately 200 bp (first peak cluster) and 1 kb (second peak cluster), with an augmentation of the first peak cluster observed in group Y15, showing a distinct eccDNA distribution in senescent BMSCs (Figure 1B). The vast majority of eccDNAs are derived from autosomal regions, with only a few coming from sex chromosomes (Figure 1C). The distribution of split reads supporting eccDNA exhibited a consistent pattern across all four samples. The median count of these split reads reached up to 10 (Figure 1D). Notably, the location of eccDNA varied among cells at different stages of growth, correlating with prior research on eccDNA in cancer (Noer et al., 2022). Analyzing the eccDNA distribution region on chromosome 4 revealed that certain eccDNA elements were present in all samples but with varying frequencies, while others were unique to either group Y5 or group Y15 cells (Figure 1E). The mapping of all eccDNAs across the genome revealed their extensive distribution (Figure 1F).

### Genomic annotation of eccDNAs of BMSCs

**Figure 2:**
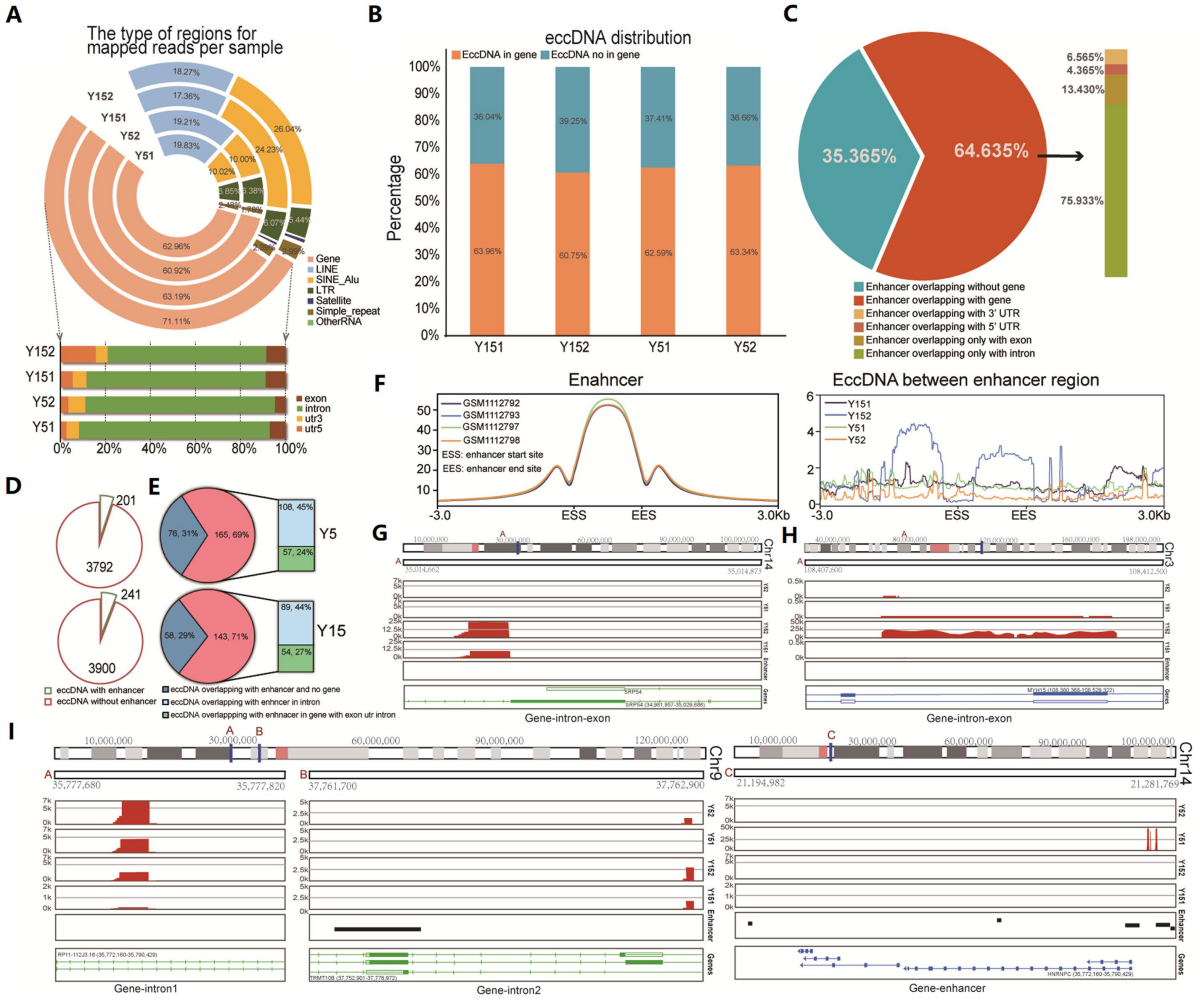
Distribution of eccDNA in different genomic element regions. **(A)** Distribution of eccDNA reads in the genome region revealed their various genomic locations, including the gene region, LINE region, SINE_Alu region, LTR region, satellite region, simple repeat, and others. **(B)** The bar graph shows eccDNA fragment distribution in gene and non-gene regions. **(C)** The relationship between enhancer region and gene regions. Those ChIP-seq data of H3K27ac with labeled enhancers (GSM1112792, GSM1112793, GSM1112797, and GSM1112798) were downloaded, and the distribution of all enhancers was analyzed. **(D)** The pie chart shows the overlap between eccDNA and MSC enhancers in the Y5 and Y15 groups. **(E)** Genomic distribution of eccDNAs that overlapped with enhancers. **(F)** The distribution of enhancers and the relationship between enhancers and eccDNA location. **(G–I)** Chromosomal location of eccDNAs.

To investigate the potential functional role of eccDNA, we conducted a comprehensive genomic distribution analysis. We first carefully examined the total mappable reads associated with eccDNA, classifying them into distinct genomic elements such as gene regions, satellite regions, simple repeat regions, LINEs, SINEs, and other RNA (Figure 2A). The fraction of reads mapped to each genomic element class relative to the total valid genome reads was computed. Notably, eccDNA reads came from a variety of genomic sites, including both genic and intergenic regions. We found an enrichment inside gene intronic regions in particular (Figure 2A). We discovered that over 60% of eccDNA fragments were distributed within genes or overlapped with gene areas when deeply predicting from eccDNA readings (Figure 2B). Introns, which are known to alter gene transcription rates by acting as regulatory elements such as enhancers and repressors (Le Hir et al., 2003), appear to be crucial in this situation. Previous research and review have shown that eccDNAs can be an effective strategy for gene amplification, either by directly boosting copy number or by serving as trans-acting factors such as super-enhancers (deCarvalho et al., 2018; Xu et al., 2019). We collected enhancer area data for MSC from publicly available ChIP-seq datasets (GSM1112792, GSM1112793, GSM1112797, and GSM1112798) and evaluated its genome distribution using the H3k27ac antibody, a modification suggestive of enhancer activity. Surprisingly, the majority of enhancers (64.635% of the total) were positioned within gene intervals or overlapped with genes, with 75.933% of these enhancers placed within intronic regions (Figure 2C). Subsequently, we conducted a detailed analysis of the spatial relationship between eccDNA and MSC enhancers. This research found that 201 eccDNAs in the Y5 group overlapped with enhancers, while 3,792 eccDNAs had no overlap with enhancers. Similarly, 241 eccDNAs in the Y15 group overlapped with enhancers, but 3,900 eccDNAs did not (Figure 2D). Notably, 69 and 71% of eccDNAs overlapping with enhancers were discovered to be situated within genes in the Y5 and Y15 groups, respectively, with the Y5 group exhibiting an intronic eccDNA ratio of 45% and the Y15 group displaying 44% within intronic areas (Figure 2E). Furthermore, the enhancer area showed a strong peak at the enhancer locus, with lesser peaks both upstream and downstream, but there was no discernible pattern in the distribution of eccDNAs obtained from MSC for the enhancer region (Figure 2F). Furthermore, chromosomal mapping of eccDNAs revealed that they were distributed across the genome in intronic or untranslated regions (Figures 2G–I). In conclusion, our data imply that eccDNAs may not operate primarily as enhancers in grown MSC.

### RNA-seq revealed senescence-related gene expression in cultivated BMSCs

**Figure 3:**
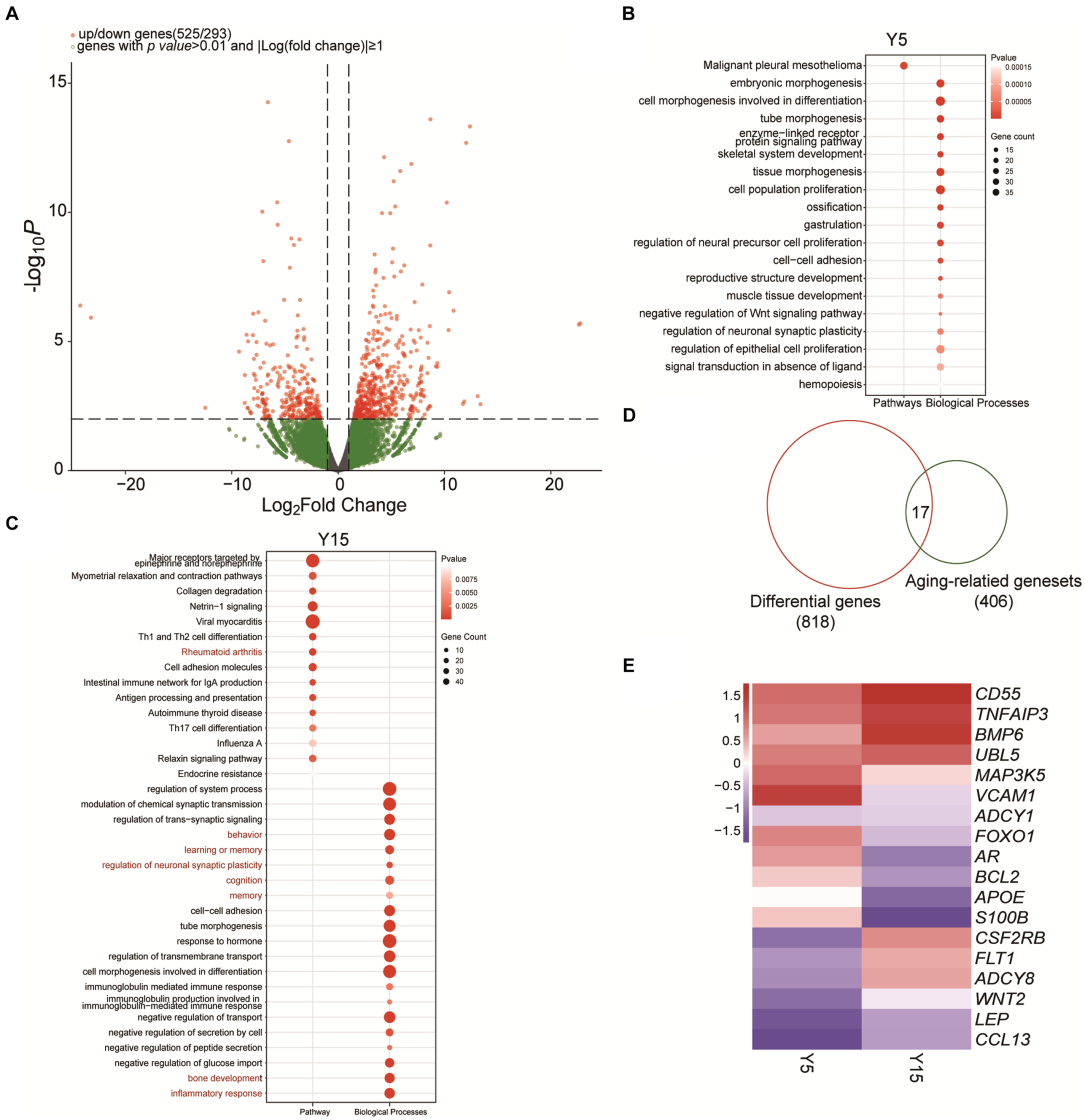
Functional analysis of differentially expressed genes and identification of senescence-related genes. **(A)** Volcano plot of significantly differentially expressed genes between the Y5 and Y15 groups. **(B,C)** GO and KEGG enrichment analysis results of upregulated genes in the Y5 and Y15 groups. **(D)** Overlap analysis between senescence-related genes from the National Aging Database and DEGs. **(E)** Clustered heatmap of differentially expressed senescence-related genes in Y5 and Y51 groups. Red, upregulation; blue, downregulation.

We investigated the effect of extended culturing on gene expression in BMSC cell lines in depth. We detected 525 substantially upregulated mRNAs and 293 significantly downregulated mRNAs (*p-value* ≤ 0.01, |Log fold change| ≥ 1) in the Y5 and Y15 groups using rigorous RNA-sequencing analysis (Figure 3A). Following that, these differentially expressed genes (DEGs) were subjected to a thorough biological process and enrichment analysis. Surprisingly, cell morphogenesis related to differentiation, cell population proliferation, and hemopoiesis were the major functional modules among upregulated candidates in the Y5 group (Figure 3B). The upregulated DEGs in the Y15 group, on the other hand, were enriched in processes such as system function regulation, collagen degradation, rheumatoid arthritis, neuronal synaptic plasticity regulation, inflammatory response, and immune cell morphogenesis involved in Th17 cell differentiation (Figure 3C).

EccDNA has long been implicated in the senescence process of yeast, as well as mammalian cells and tissues (Hull et al., 2019). We found 17 senescence-related genes that exhibited significant differential expression in our study by combining 406 senescence-related genes from the National Senescence Database with the 818 discovered DEGs (Figure 3D). Notably, the Y15 group had higher levels of Adenylyl Cyclase Type 8 (ADCY8), VEGF-specific membrane receptors (FLT1), bone morphogenetic protein (BMP6), and GM-CSF receptor (CSF2RB) expression than the Y5 group (Figure 3E). In contrast, the Y15 group had lower levels of vascular cell adhesion molecule-1 (VCAM-1), FOXO1, and androgen receptor (AR) (Figure 3E).

### eccDNAs are the potential binding site of TFs

**Figure 4:**
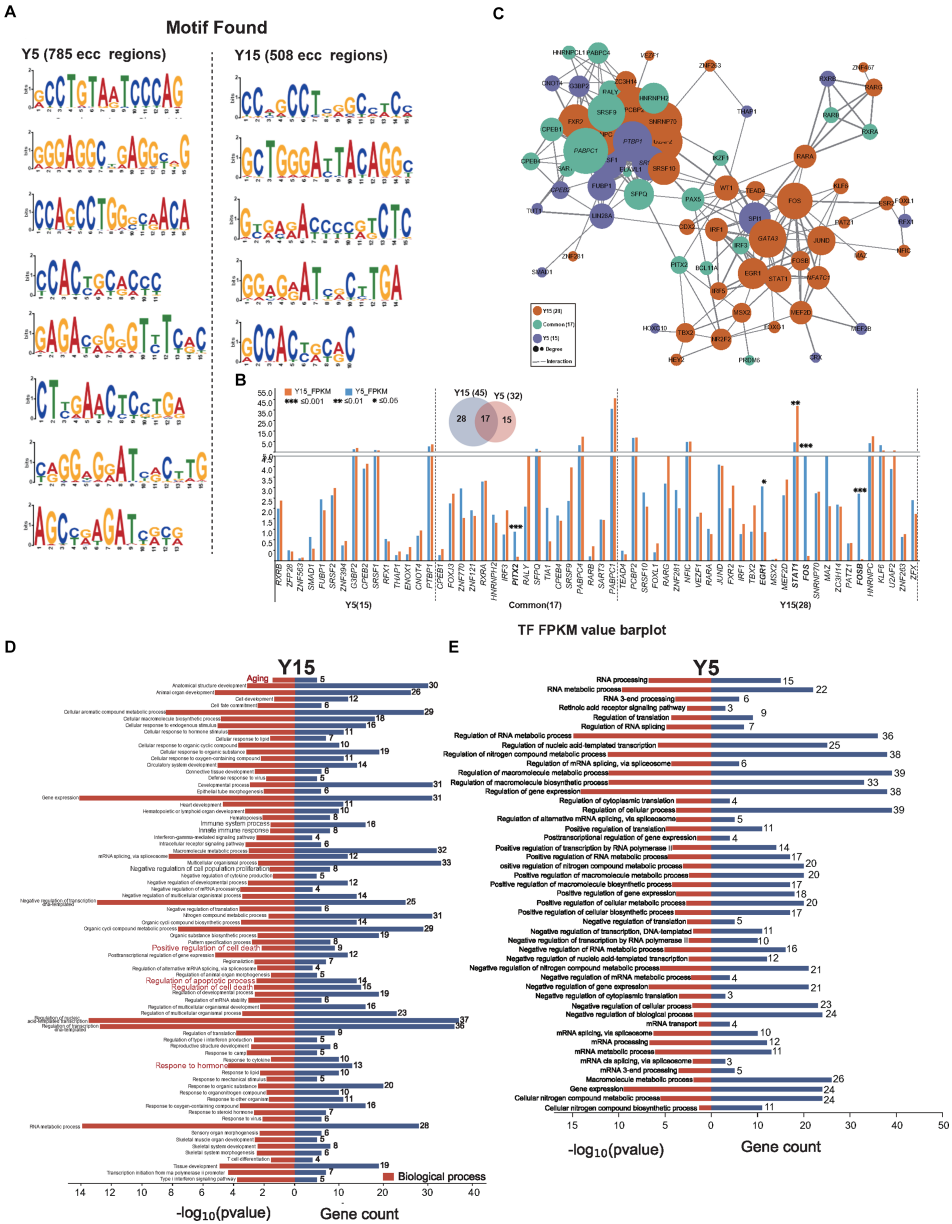
EccDNA affects TF-binding pathways. **(A)** Motif scan in the Y51 group and Y15 group. **(B)** The bar plot uncovers the expressional difference of known TF binding to the enriched motif context as above. **(C)** The PPI network relationship between found known TFs. **(D,E)** GO analysis found TFs in the Y15 and Y5 groups, respectively.

Intricate motif sequence in genome-wide spread often harbors specialized functions, such as mediating protein interactions or orchestrating specific TFs to regulate target gene expression (Bader and Hogue, 2003; Traut, 2005). Using the MEME suite web tool (Bailey et al., 2015), we performed motif analysis on eccDNA sequences to investigate the function of eccDNA in the culture of BMSCs. We identified 8 enriched motifs in 785 eccDNAs from the Y5 group and 5 enriched motifs in 508 eccDNAs from the Y15 group (Figure 4A). These motifs often operate as binding sites for particular proteins, such as TFs, which then control the regulation of related genes. Upon closer examination, motif-binding TF proteins were found in every group. As illustrated in Figure 4B, 32 TFs emerged in the Y5 group and 46 in the Y15 group, with a notable overlap of 17 TFs between the groups. Notably, there was a considerable overlap of 17 TFs between the groups. Remarkably, 5 of the 61 TFs showed significant variation in gene expression levels; these five TFs were all in the Y15 group (Figure 4B). An extensive web of physical interactions within specific protein sets was revealed by protein–protein interaction (PPI) network research. This result implied that these TFs carry out their activities through mutual contacts, either by joining forces to create protein complexes or by working in tandem (Figure 4C). Furthermore, major biological processes associated with senescence, such as positive regulation of cell death, apoptotic processes, and regulation of cell death, were identified by GO analysis of TF-regulated genes in the Y15 group. The pathways found in the Y5 group, on the other hand, included RNA processing, mRNA transport, and control over cytoplasmic translation (Figures 4D,E).

**Figure 5:**
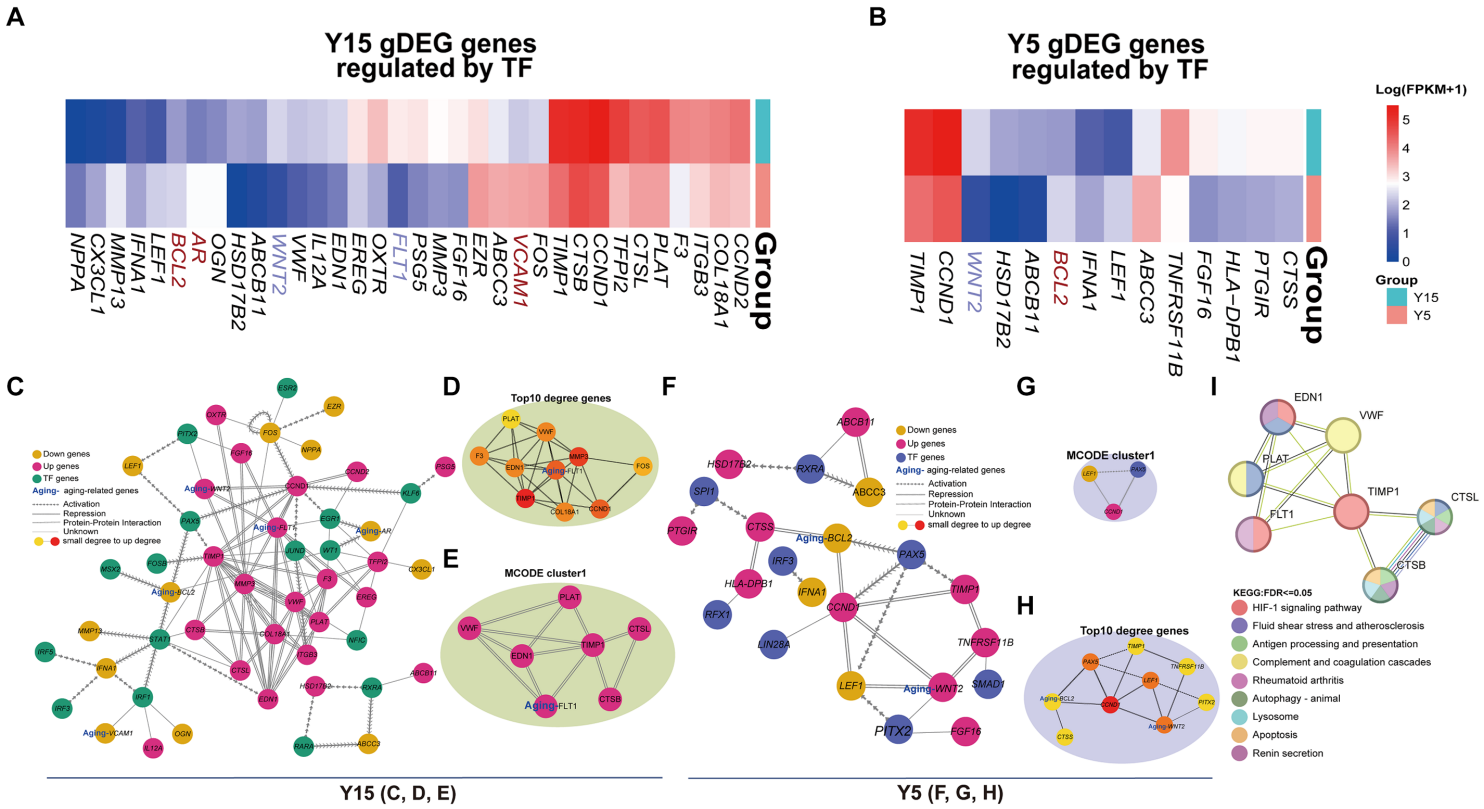
EccDNA affects the TF-binding network. **(A,B)** The heatmap shows the expression of DEGs regulated by TFs above in the Y15 and Y5 groups, respectively. **(C–E)** The comprehensive and pivotal network relationship between found TFs and corresponding DEGs in the Y15 group. **(F–G)** The comprehensive and pivotal network relationship between found TFs and corresponding DEGs in the Y5 group. **(H)** The top 1 sub-network based on MCODE and relative KEGG annotation.

The understanding of TF function and its impact on gene expression is dependent on the discovery of TF-target regulatory connections. By searching TRRUST for TF-regulated genes, we discovered 34 DEGs in the Y15 group and 14 DEGs in the Y5 group that were controlled by TFs from corresponding motifs (Figures 5A,B). Three senescence-related genes were discovered in the DEGs of the Y15 group and one in the Y5 group. Combining PPI networks and putative co-regulations for specific DEGs and TFs (Figures 5C–H) highlighted the mutual regulatory linkages and distinctions between the two groups. Furthermore, the Y15 group had a distinct functional module enriched with genes and TFs linked with senescence, which was conspicuously absent in the Y5 control group. This module exhibited a significant level of enrichment in interactions, comprising the bulk of senescence-related genes and TFs. Fluid shear stress and atherosclerosis (Shaaban and Duerinckx, 2000), complement and coagulation cascade (Anderson et al., 2010), rheumatoid arthritis (Serhal et al., 2020), apoptosis, and renin secretion (Bauer, 1993; Figure 5I). These findings provide compelling support for the central role of eccDNA in shaping the genetic landscape during BMSC cultivation, ultimately governing cellular senescence.

## Discussion

The senescence of stem cells increases in tandem with the increasing functional deficits that characterize aging (Lei et al., 2021). Although the involvement of MSCs in senescence-related disorders and cellular treatment has been proposed clinically, there are barriers to their application (Al-Azab et al., 2022). *Ex vivo* growth of MSCs, for example, promotes oxidative stress and cellular senescence, resulting in poor *in vivo* engraftment and functions (Denu and Hematti, 2016; Yoon et al., 2022). Premature senescence of MSCs, in particular, represents a key issue for clinical applications, as senescence MSCs adopt a senescence-associated secretory phenotype, which alters the therapeutic effectiveness (Al-Azab et al., 2022). To utilize the regenerative potential of cultivated MSCs for therapeutic therapies, cellular senescence must be prevented, and the mechanisms behind cellular senescence must be understood. We expanded the current understanding of MSC senescence at the eccDNA level and reported the first eccDNA profiles of young and senescence BMSCs since genomic mistakes constitute the first “on switch” of MSC senescence and eccDNA synthesis is dependent on DNA organization and damage repair (Kundrotas et al., 2016; Paulsen et al., 2018). Furthermore, we revealed that eccDNA can act as a TF-binding motif to regulate senescence-related gene expression in BMSCs in an enhancer-independent manner, and the genetic properties of eccDNAs may point to a novel route for therapeutic therapy of senescence-related disorders.

Previous studies have found that eccDNA levels may significantly increase during the human senescence process (Qiu et al., 2021). EccDNA sequencing has a wide variety of applications in human senescence. The entire understanding of the composition, structure, and function of eccDNA was identified by sequencing to provide support for the prevention and therapy of aging-related illnesses such as cancer and cardiovascular disease. Hull et al. (2019) proved that the accumulation of the copper-resistance gene *CUP1* eccDNA is related to age-linked genetic change. Importantly, the eccDNAs discovered in this study may be produced from any genome and are roughly proportionate to the overall abundance of gene and non-gene sections in the genome, as previously reported (Wu et al., 2022). Previous research has demonstrated that the size distribution of eccDNA ranges from 10’s to 100’s of 1,000’s of base pairs in examined eukaryotes, including normal human tissues, cancer, plasma, and serum (Møller et al., 2020; Gerovska and Arauzo-Bravo, 2023). We found abundant eccDNAs in BMSCs ranging in size from 0.01 to 1,000 kb, with the majority ranging from 0.1 to 10 kb and exhibiting two different peaks approximately 300 bp and 1 kb. Furthermore, the length of eccDNAs in senescent BMSCs was shorter than in youthful cells, which could be attributed to changes in the senescence microenvironment. The highest density of eccDNA distribution, on the other hand, was discovered on chromosome 1, which is the longest chromosome (Hwang et al., 2013). The least amount of eccDNA was observed on chromosome Y, which has a low gene density. Moreover, certain eccDNA segments appear differently in young and senescence BMSCs, indicating their role during MSC senescence. When we mapped eccDNA to different types of genomic elements to study their formation preferences, we found that eccDNA was most abundant in intron regions. The pattern found here differed from that previously reported in the literature for eccDNAs in mice, human plasma, and cancer cells (Zhu et al., 2022), probably due to the distinct cell type; however, the specific explanation remains unknown. We hypothesized that eccDNA might interact with enhancers to regulate senescence-related gene expression in BMSCs because transcriptional regulation elements such as enhancers and repressors were always distributed in the intron region and eccDNA could enhance gene amplification in various cells (Li et al., 2012; Wu et al., 2019; Molin et al., 2020). Surprisingly, overlap analysis revealed very little connection between eccDNAs and BMSC enhancers.

We performed motif analysis on the eccDNA sequences to look for potential control mechanisms of eccDNA during MSC senescence and discovered that particular patterns could enhance TF. The GO and KEGG pathway analyses of differentially enriched TFs in senescence BMSCs revealed enrichment in “aging,” “positive regulation of cell death,” “regulation of apoptotic process,” “regulation of cell death,” and “response to hormones,” all of which have been linked to cell senescence (Sui et al., 2016). Meanwhile, no senescence-related pathways were found to be enhanced in young BMSCs, implying that a particular eccDNA might be used as a BMSC senescence biomarker.

In conclusion, we first showed that senescent human BMSCs have a distinct landscape and eccDNA expression pattern when compared to young BMSCs, and eccDNA has significant potential to be employed as a therapeutic target for senescence-related disorders. Given the remarkable stability of eccDNA, future research should look into the potential of chemically produced eccDNAs as TF regulatory factors to reverse senescence.

## Data availability statement

The datasets presented in this study can be found in online repositories. The names of the repository/repositories and accession number(s) can be found in the article/Supplementary material.

## Ethics statement

Ethical approval was not required for the studies on humans in accordance with the local legislation and institutional requirements because only commercially available established cell lines were used.

## Author contributions

WY: Writing – original draft, Data curation, Formal analysis, Investigation, Methodology, Software. WJ: Data curation, Investigation, Methodology, Software, Validation, Writing – original draft. BL: Writing – original draft. ZL: Writing – original draft. JiaW: Writing – original draft. HL: Writing – original draft. JinW: Funding acquisition, Visualization, Writing – original draft, Writing – review & editing. QH: Funding acquisition, Visualization, Writing – original draft, Writing – review & editing.

## Footnotes

^2^https://cran.r-project.org/web/packages/pheatmap/index.html
