# Supplementary material for: Genome-wide sequencing identified extrachromosomal circular DNA as a transcription factor-binding motif of the senescence genes that govern replicative senescence in human mesenchymal stem cells
Source: Front Cell Neurosci. 2024 Aug 2;18:1421342. doi: 10.3389/fncel.2024.1421342 (PMC11327076; doi:10.3389/fncel.2024.1421342)
Supplement: Supplementary file 1 [file Data_Sheet_1.PDF]

9:17

贺乾

Jingbo wang

最好直接图片吧

I prefer image

images in this document is different with my original document

这个里面的和我这里的原始数据图不一样

Qian He

好的 那估计得晚上了 她还在开会

OK, but she is still in meeting,maybe later at night

第一个就不一样，后面的我懒得比了

The first figure is different
I am lazy to compare the others

那得等她搞完了 文字你可以比着word里面新图搞一下 图片就得等她了

then we have to wait for her, you can modifiy the text first and waiting for her figure

2023年12月28日 下午15:46

OK OK 嗯嗯

2023年12月28日 下午16:47

manuscriptFigurepngANDtif_.rar
39.4 MB 接收中断

微信电脑版

the supplementary figure is the redundancy results of main figure, we did not cite them in the manuscript, them?

我看附图都是和主图冗余的结果 正文里面也没引用 就不要了吧

OK 好的

10:18

李忠波

2022年3月21日 上午10:11

Qian He

eccDNA整理.zip
138.5 MB
results of eccDNA
微信电脑版

Qian He

2022年3月21日 上午10:29

Zhongbo Li

I will go to work in the afternonn, we will say it again
我下午去上班哦 下午过去说

OK
好的

我把画的图先发给你吧
I will send you the image first

你先看看
You check it first

2022年3月21日 上午10:54

OK

2022年3月21日 下午13:03

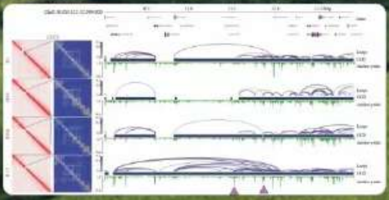

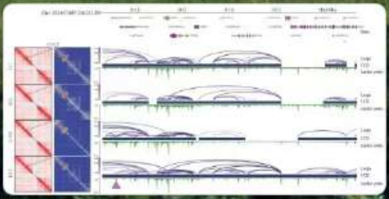

10:09

冀伟

2021年6月7日 下午14:35

Wei Ji

贺老师好，4个样品的eccDNA的 circular DNA coordinates bed文件得到了，目前是得到了前6列的信息。后面几列由于内存原因，还没算出来，但是我看说明，貌似不影响挑出结果

Afternoon, Dr He, I have obtained the circular DNA coordinated bed document of the four samples, and the first 6 information was identified. due to the internal storage, the calculation did not fished. I have read the instructions and it do not influence the results

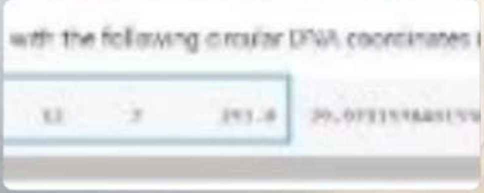

脚本分析完给了一个得到eccDNA的数目，我在整理文件，看是否可以对的上数目，因为程序得到的结果，有些坐标是重复的

I obtain the number of eccDNA through script analysis and now I am checking it, for some repeated axis.

后面的联合分析，您这边有思路嘛？

Do you have any ideas of the further analysis?

2021年6月7日 下午14:43

Qian He

好的 你整理一下 先看看mapping情况 以及数量 大小 染色质定位等基本信息 在venn做个样本之间的交互 看看样本之间共有和特有的环状DNA 如果没问题的话 你让杨巍教你一下把eccDNA和RNA的文件上传一下我们的basic browser

OK, you check the mapping situation first and the basic information of our samples. make a mapping analysis between samples to confirm if there are any special dccDNA. It there is no more problem,you can ask Yang Wei to teach you upload eccDNA and RNA ducuments to our basic browser

嗯嗯 这4个eccDNA样品 也是 [illegible]

10:08

冀伟

2021年6月23日 上午09:39

Wei Ji

贺老师可以提供一个样品的eccDNA的文件给我看看吗?

Dr He，could you show me one of the eccDNA documents ?

我想看看两个文件的位置差异是怎么样的

I want to check the location difference between documents

如果方便的话

If it is convenient

Qian He

bam 文件没释放 我今天才发现

I just notice that the Bam document did not release

有 excel 吗?

any excel?

有

yes

那我先看看坐标

then I check the axis first

The circular DNA was detected by the Circle-Map.具体方法和参数和 circlemap 默认的一样，您可以看下 https://github.com/iprada/Circle-Map

detailed methods and parameter are the same as circlemap, you can visit.........

这是公司回复的

the company replied

我用的也是这个网页的软件

I also use the software form this website

10:08

冀伟

2021年7月21日 中午12:29

Wei Ji

贺老师，我的网页的账户权限遇到一些问题，杨巍还在帮我处理，等处理好我会把转录组和eccDNA的bedgraph都传上去

Teacher He, something went wrong with my account authority, Yang Wei still handling it for me. I will upload the RNA and eccDNA bedgraph.

Qian He

好的

OK

2021年7月23日 下午15:51

方便电话吗

convenient to answer the phone?

2021年7月23日 下午16:00

嗨，我刚看到消息

just seen this message

方便

with pleasure

ok

2021年7月23日 下午16:06

聊天时长 04:53

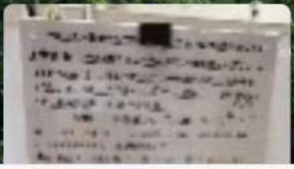

9:49

杨巍

Qian He

通话时长 05:50

Wei Yang

2023年6月29日 上午11:57

Figure3_v9.pdf
915.8 KB
PDF
微信电脑版

Figure5abcde_v8.pdf
4.0 MB
PDF
微信电脑版

Fig2abcde_v7.pdf
1.6 MB
PDF
微信电脑版

那个基因的确实不好找 it is not easy to find the target gene

太难了，眼睛要看瞎了 too difficulte, I am nearly blind

找出来这么几个 find these several

先用着吧 use it first

2023年6月29日 中午12:02

OKOK 好的好的

9:48

杨巍

2023年9月15日 下午14:26

Wei Yang

manuscript updated_yw.docx
2.2 MB
微信电脑版

贺乾师兄，你看下是否可以？标砖红的是我改写的和添加的

Dr He, I have marked the changes with brick red, please check it and tell me if it is OK.

Qian He

好的

OK

方法部分的需要添加的实验的部分的，我用黄色的标注了下，因为那个实验我不知道具体怎么做的，没办法加。

I marked the experiment in the method part with yellow, as I am not sure the detailed procedure.

OK 我看看

OK, I will chechk it

方法部分的我看了下，还是之前写的那些，因为之前也是按照文章中的方法写的，所以就做太大的修改

I have read the previous written methods, as they were corresponding with references, there were big changes.

就没做太大的修改，我个人觉得是没啥问题的

there were no big changes and I think it is no problem

你看下，如果要再修改的，你再告诉我

you can read this first and tell me if need further modification

好的

OK

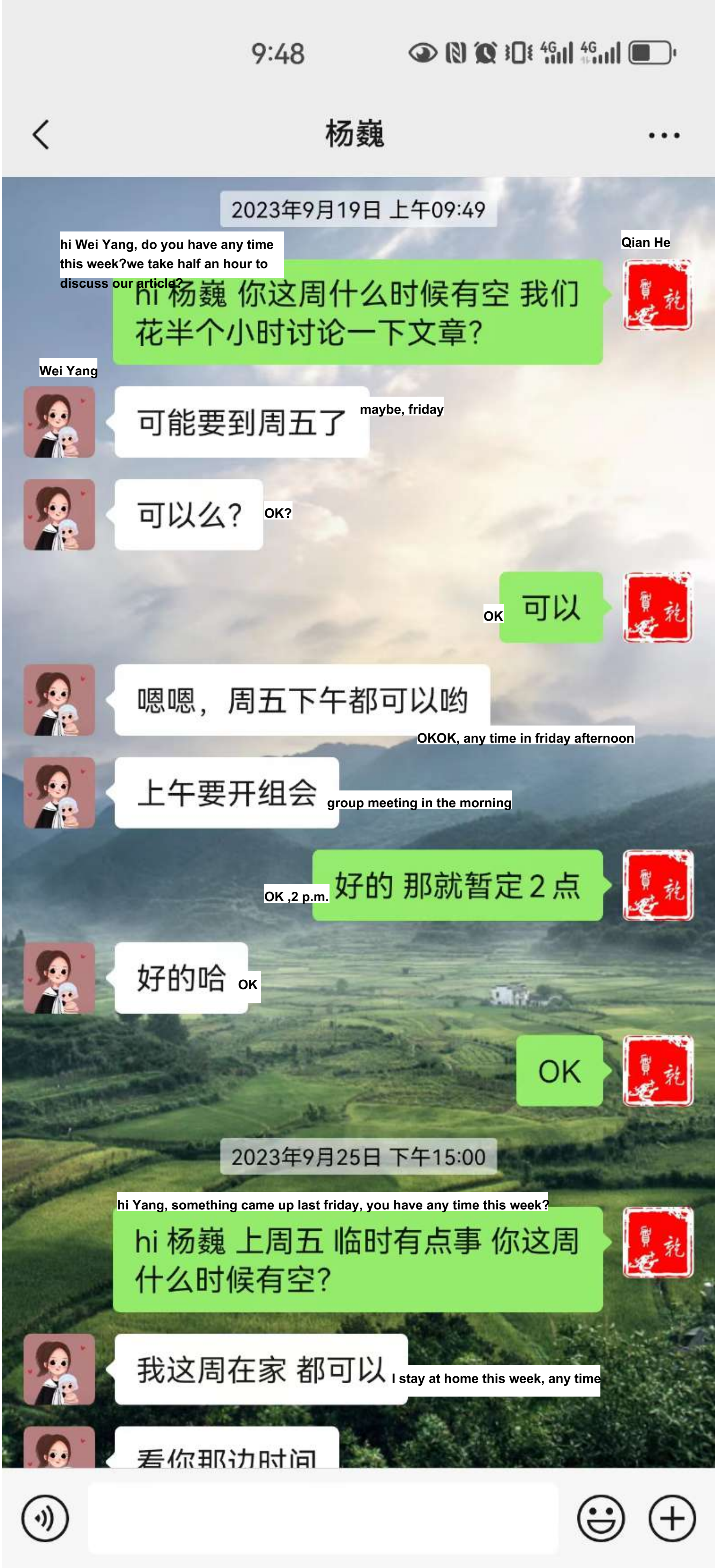

9:48
杨巍
2023年9月19日 上午09:49
Qian He
hi Wei Yang, do you have any time this week?we take half an hour to discuss our article?
hi 杨巍 你这周什么时候有空 我们花半个小时讨论一下文章?
Wei Yang
可能要到周五了
maybe, friday
可以么?
OK?
可以
OK
嗯嗯，周五下午都可以哟
OKOK, any time in friday afternoon
上午要开组会
group meeting in the morning
好的 那就暂定 2 点
OK ,2 p.m.
好的哈
OK
OK
2023年9月25日 下午15:00
hi Yang, something came up last friday, you have any time this week?
hi 杨巍 上周五 临时有点事 你这周什么时候有空?
我这周在家 都可以
I stay at home this week, any time

9:34

廖柏瑜 23研究生

Boyu Liao

不然我还是找个时间和老师讨论一下我的课题的后续可以嘛 I want to discuss my further task with teacher sometime.

now? 现在?

Jingbo Wang

2月23日 上午09:21

不是 no

下星期吧 next week

OK 可以

我现在复苏的细胞长的还有点慢

My resuscitated cell grow a little slow

嗯嗯，谢谢老师 OKOK, thanks teachter

It will grow better after several passages 传几代就好了

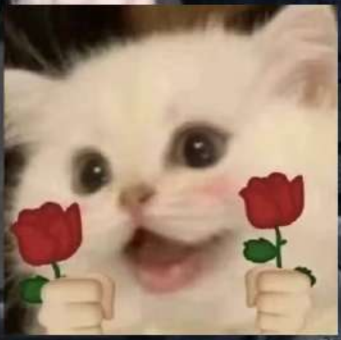

是的是的 yes

2月23日 上午11:02

[illegible]

9:21

廖柏瑜 23研究生

2023年11月22日 上午10:52

PYGL_PYJH_YJSGRPYJH_SZU.pdf
187.4 KB
PDF

2023年11月22日 下午16:37

在我桌上

2023年11月22日 下午17:46

说到，老师

2023年11月23日 上午09:19

你今天有空的时候，再帮我登录一下这个发两个稿

青衣：网址：https://www.sztu.edu.cn:8443/system/login.jsp 账号：y00616...

好的好的老师，我中午下课吃完饭后回宿舍弄一下！

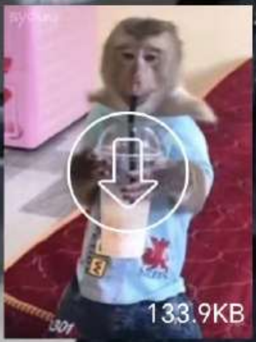

嗯嗯

9:17

贺乾

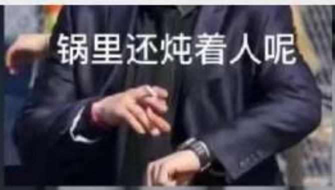

Qian He

应该差不多

it is OK

2023年12月11日 上午10:18

Jingbo Wang

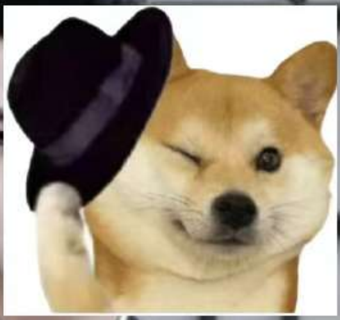

2023年12月12日 下午17:30

manuscript updated_yw_v220231211.docx
877.7 KB
微信电脑版

你再看看

you check this version again

🙏

2023年12月12日 下午17:36

OK 好

2023年12月27日 上午11:03

把你写成我们研究生的校外导师

10:01

王坚

2021年4月15日 上午09:56

Jian Wang

贺老师，eccDNA的分析和数据，邮件发您了 Dr He, the analyzed data of eccDNA has e-mailed to you

记得查收哈 remember to check you email

Qian He

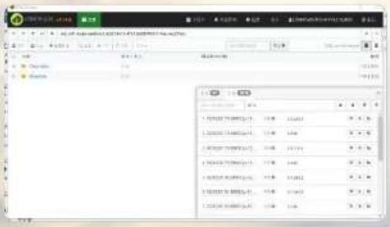

The original data can not be downloaded

这个原始数据一下载就显示失败

我问一下 I will ask why

OK 好的

2021年4月15日 上午10:13

是网络原因。数据量太大，重新下载一下。 network problem, its too big, download it again

OK 好的

2021年4月15日 下午14:25

Dr He，any more problem on data download

贺老师，数据下载可以了吗

好像是网络问题 但是下到一半就又
