# Supplementary figures and images for: Genome-wide sequencing identified extrachromosomal circular DNA as a transcription factor-binding motif of the senescence genes that govern replicative senescence in human mesenchymal stem cells
Source: Front Cell Neurosci. 2024 Aug 2;18:1421342. doi: 10.3389/fncel.2024.1421342 (PMC11327076; doi:10.3389/fncel.2024.1421342)

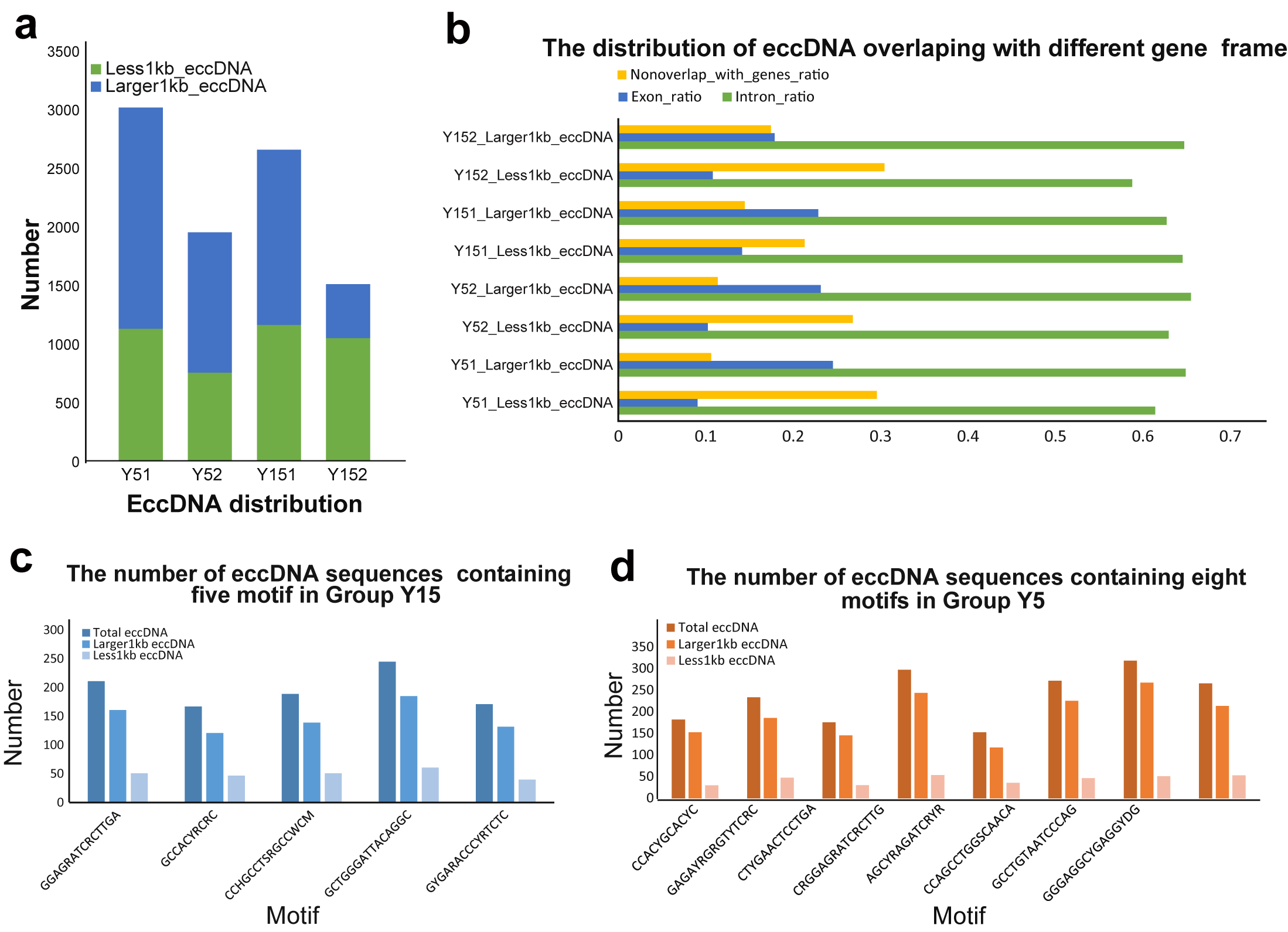

Supplement: Supplementary file 3 [file Image_1.TIF]
